# Mobile fosfomycin resistance genes in Enterobacteriaceae—An increasing threat

**DOI:** 10.1002/mbo3.1135

**Published:** 2020-10-30

**Authors:** Katrin Zurfluh, Andrea Treier, Kira Schmitt, Roger Stephan

**Affiliations:** ^1^ Institute for Food Safety and Hygiene Vetsuisse Faculty University of Zurich Zurich Switzerland

**Keywords:** antimicrobial resistance, dissemination, Enterobacteriaceae, transferrable fosfomycin resistance

## Abstract

Antimicrobial resistance is one of the major threats to the health and welfare of both humans and animals. The shortage of new antimicrobial agents has led to the re‐evaluation of old antibiotics such as fosfomycin as a potential regimen for treating multidrug‐resistant bacteria especially extended‐spectrum‐beta‐lactamase‐ and carbapenemase‐producing Enterobacteriaceae. Fosfomycin is a broad‐spectrum bactericidal antibiotic that inhibits the initial step of the cell wall biosynthesis. Fosfomycin resistance can occur due to mutation in the drug uptake system or by the acquisition of fosfomycin‐modifying enzymes. In this review, we focus on mobile fosfomycin‐resistant genes encoding glutathione‐S‐transferase which are mainly responsible for fosfomycin resistance in Enterobacteriaceae, that is, *fosA* and its subtypes, *fosC2*, and the recently described *fosL1*‐*L2*. We summarized the proposed origins of the different resistance determinants and highlighted the different plasmid types which are attributed to the dissemination of fosfomycin‐modifying enzymes. Thereby, IncF and IncN plasmids play a predominant role. The detection of mobile fosfomycin‐resistant genes in Enterobacteriaceae has increased in recent years. Similar to the situation in (East) Asia, the most frequently detected fosfomycin‐resistant gene in Europe is *fosA3*. Mobile fosfomycin‐resistant genes have been detected in isolates of human, animal, food, and environmental origin which leads to a growing concern regarding the risk of spread of such bacteria, especially *Escherichia coli* and *Salmonella*, at the human–animal–environment interface.

## Graphical abstract

The shortage of new antimicrobial agents has led to the re‐evaluation of old antibiotics such as fosfomycin as a potential treatment regimen against multidrug‐resistant bacteria, especially extended‐spectrum‐beta‐lactamase‐ and carbapenemase‐producing Enterobacteriaceae. In Enterobacteriaceae, gene‐encoding fosfomycin‐modifying enzymes are frequently found on plasmids, transposons or within integrons enabling rapid dissemination. There is growing concern regarding the risk of spread of such bacteria, especially *Escherichia coli* and *Salmonella*, at the human–animal–environment interface.

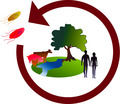

## 1 INTRODUCTION

Fosfomycin, originally known as phosphonomycin, was discovered five decades ago and has been approved for the treatment of uncomplicated urinary tract infections (UTI) since early 1970 (Silver, 2017). The phosphonic acid‐derived antibiotic containing an epoxide and a propyl group possesses a unique chemical structure and the mechanism of action comprising broad‐spectrum activity against Gram‐negative and Gram‐positive aerobic bacteria is unrelated to any other antibiotic family (Castañeda‐García et al., 2013; Silver, 2017). Fosfomycin has a bactericidal effect by inhibiting the initial step in the biosynthesis of peptidoglycan. It acts as a phosphoenolpyruvate (PEP) analogue and binds the essential enzyme MurA (UDP‐*N*‐acetylglucosamine enolpyruvyl transferase) leading to bacterial cell lysis and death (Castañeda‐García et al., 2013; Kahan et al., 1974).

The shortage of new antimicrobial agents has led to the re‐evaluation of old antibiotics such as fosfomycin and colistin as potential treatment options against multidrug‐resistant (MDR) bacteria especially extended‐spectrum‐beta‐lactamase‐ and carbapenemase‐producing Enterobacteriaceae, that is, the use of fosfomycin in other indications as uncomplicated UTI and potentially in combination with other antibiotics (Falagas et al., 2010; Samonis et al., 2012; Silver, 2017).

Three major fosfomycin resistance mechanisms have been described: (a) reduced permeability to fosfomycin, (b) modification of the antibiotic target MurA, and (c) antibiotic modification, whereas reduced drug uptake remains the most frequently encountered resistance mechanism among clinical isolates as well as in *in vitro* obtained mutants (Castañeda‐García et al., 2013; Nilsson et al., 2003; Silver, 2017). In *Escherichia coli*, this chromosomally encoded resistance mechanism is based on mutations in two main nutrient transport systems which are responsible for the fosfomycin uptake, namely the glycerol‐3‐phosphate transporter (GlpT) and the glucose‐6‐phosphate transporter (UhpT) (Kahan et al., 1974). In general, modification of the antibiotic target is one of the most common mechanisms to acquire antibiotic resistance in bacteria. However, modifications of MurA seem to be rare in clinical isolates. Nevertheless, it has been shown that overexpression of the *murA* gene contributes to the acquisition of clinical levels of fosfomycin resistance at low fitness cost (Couce et al., 2012). These chromosomal resistance mechanisms have been recently reviewed in detail (Castañeda‐García et al., 2013; Silver, 2017).

Meanwhile, several fosfomycin‐modifying enzymes have been characterized that lead to the inactivation of the drug. Fosfomycin‐producing microorganisms such as some strains of *Streptomyces* and *Pseudomonas syringae* exhibit antibiotic kinases (FomA or FomB) that modify and detoxify the antibiotic inside the cell by phosphorylation (Kobayashi et al., 2000). In clinical important pathogenic bacteria, fosfomycin resistance involves metalloenzymes that catalyze the opening of the epoxide (oxirane) ring by adding glutathione (FosA1‐FosA10, FosL1‐2, FosC2), bacillithiol (FosB), or water (FosX) (Rigsby et al., 2005). FosB (Mg^2+^‐cofactor) type enzymes are found in low GC Gram‐positive bacteria such as *Bacillus subtilis*,*B*.* anthracis*, *Staphylococcus epidermidis*, and *S*.* aureus*. FosX (Mn^2+^‐cofactor) has been described in Gram‐positive bacteria such as *Listeria monocytogenes*,*Clostridium botulinum*, and *Brucella melitensis* (Silver, 2017). In this review, we will focus on *fosA* and its subtypes, *fosC2*, and the recently described *fosL1*‐*L2* since these genes are mainly responsible for fosfomycin resistance in Enterobacteriaceae.

In Enterobacteriaceae, genes encoding fosfomycin‐modifying enzymes are frequently found on plasmids, transposons, or within integrons. Regardless of the location (chromosomal or plasmid), insertion sequences are often found in proximity indicating mobilization of these genes. These facts enable rapid dissemination and indeed, fosfomycin‐modifying enzymes are widespread in Asia where most of these genes initially have been described. Moreover, plasmids carrying *fos*‐genes commonly harbor additional resistance genes that foster the chance of co‐selection of fosfomycin resistance under the selective pressure by other antibiotics (Poirel et al., 2018).

The efficacy of fosfomycin against extended‐spectrum beta‐lactamase (ESBL)‐producing Enterobacteriaceae is favorable although increasing fosfomycin resistance rates due to fosfomycin‐modifying enzymes have been observed among ESBL‐producing and carbapenemase‐producing Enterobacteriaceae in the last decade, primarily in Asia but more and more globally (Baron et al., 2020; Benzerara et al., 2017; Chan et al., 2014; Fernandes et al., 2018; Ho, Chan, Lo, Lai, et al., 2013; Hou et al., 2012; Wachino et al., 2010).

Co‐selection and frequent use of fosfomycin are important factors in resistance development and dissemination. Fosfomycin is approved for use against uncomplicated UTI almost all over the world. In some European countries, that is, Spain, France, Germany, Austria, and Greece, the intravenous formulation is administered in combination with other antibiotics for the treatment of nosocomial infections due to multidrug‐resistant (MDR) Gram‐positive and Gram‐negative bacteria (Michalopoulos et al., 2011). In contrast, fosfomycin is not approved for veterinary use in most countries including China and Europe (Wang et al., 2017). However, fosfomycin is widely used in farms in Argentina, Brazil, and Central America, primarily for the treatment of infectious diseases in broiler chickens and pigs (Pérez et al., 2014).

## 2 ORIGIN OF *FOSA* GENES

Ito and colleagues systematically surveyed the presence and distribution of the *fos*A genes in over 18,000 published genome sequences from 18 Gram‐negative species (Ito et al., 2017). They propose the chromosomal location of *fosA* in the genomes of *Providencia stuartii*, *Klebsiella pneumoniae*, *Serratia marcescens*, *Pseudomonas aeruginosa*, *Enterobacter aerogenes*, *Klebsiella oxytoca*, *Morganella morganii*, *Providencia rettgeri*, and *Enterobacter cloacae* based on the high prevalence of *fosA (*>80%) in the respectively analyzed genomes. In contrast, *fosA* was rarely (<5%) detected in *E*.* coli*, *Citrobacter freundii*, and *Acinetobacter baumannii* which indicated a possible acquisition of the gene. Furthermore, they could show an extensive FosA sequence diversity both within and between species. Noteworthy, key residues in the active site of the enzyme, as well as residues involved in fosfomycin binding, are highly conserved across the different FosA proteins (Castañeda‐García et al., 2013; Ito et al., 2017; Poirel et al., 2019).

Since the nomenclature of *fosA* has lacked consistency, it is proposed to distinguish between intrinsic versus acquired and chromosomal versus plasmid‐mediated origin, respectively (Ito et al., 2017). Thus, chromosomal or intrinsic *fosA*/FosA should be distinguished by adding the initials of the species whereas the numbering scheme should be reserved for acquired *fosA*/FosA (Ito et al., 2017).

The origins of plasmid‐mediated FosA alleles are summarized in Table 1. The first plasmid‐mediated *fosA* gene, *fosA1* (also known as *fosA* ^Tn^ *^2921^*), was described in a clinical isolate in Spain. *FosA1* was located within Tn*2921* on plasmid pSU912 in *S*.* marcescens* (Navas et al., 1990). FosA1 is up to 100% identical with Fos^EC^, the fosfomycin‐modifying enzyme found in the chromosome of *E*.* cloacae* which is the likely origin of FosA1 (Ito et al., 2017). Importantly, plasmid‐mediated FosA1 is distinct from FosA^SM^ which is generally present in the *S*.* marcescens* chromosome.

**TABLE 1:** Origin of plasmid‐mediated FosA alleles

FosA allele	Possible chromosomal origin	Accession no.	Reference
FosA1 (FosA^Tn2921^)	*Enterobacter cloacae*	M85195.1	Navas et al. (1990)
FosA3	*Kluyvera georgiana*	AB522970.2	Wachino et al. (2010)
FosA4	*Kluyvera georgiana*	AB908992.1	Nakamura et al. (2014)
FosA5 (FosKp96)	*Klebsiella pneumoniae*	KP143090	Ma et al. (2015)
FosA6	*Klebsiella pneumoniae*	KU254579.1	Guo et al. (2016)
FosA8	*Leclercia decarboxylata*	MN150127	Poirel et al. (2019)
FosA9	*Klebsiella variicola*	PRJEB32329	ten Doesschate et al. (2019)
FosA10	*Klebsiella pneumoniae*	MT074415	Huang et al. (2020)
FosL1	Unknown	MN464149	Kieffer et al. (2020)
FosL2	Unknown	SAMN11027629	Kieffer et al. (2020)
FosC2	Unknown	AB522969	Wachino et al. (2010)

The origin of FosA3, the most widely disseminated acquired fosfomycin‐modifying enzyme, was speculated to be *K*.* pneumoniae* after early reports (Lee et al., 2012; Wachino et al., 2010). The result of a more recent study showed that FosA3 is most closely related to FosA^KG^ (>94% identity) from *Kluyvera georgiana* among known chromosomally encoded FosA (Ito et al., 2018). Furthermore, surrounding sequences were highly similar in *K*.* georgiana* YDC799 to corresponding regions of several plasmid‐mediated *fosA3* of *E*.* coli* (Ito et al., 2017). IS*26* seems to be the key element in the mobilization of *fosA3* since it commonly composes a composite transposon surrounding *fosA3* by two IS*26* elements oriented in the opposite direction. The same IS*26*‐composite transposons frequently contain additionally a *bla*_CTX‐M_ gene (Yang et al., 2019). However, various truncations in the *fosA3* surrounding regions by IS*26* and other IS elements may give evidence for multiple independent mobilization events (Ito et al., 2018; Yang et al., 2019). The origin of FosA4, which shares 94% amino acid identity with FosA3, was similarly traced back to *K*.* georgiana* (Rodriguez et al., 2018).

The highly similar *fosA5*,*fosA6*, and *fosA10* genes (less than 10 nucleic acid sequence differences) were proposed to originate from the *K*.* pneumoniae* chromosome (Guo et al., 2016; Huang et al., 2020; Ma et al., 2015). In all three genes, *lysR* genes truncated by IS*10* were present upstream of the *fosA* gene. The downstream fragments of *fosA5*,*fosA6*, and *fosA10* varied thus separate mobilization events of *fosA* ^KP^ were likely (Guo et al., 2016; Huang et al., 2020; Ma et al., 2015).

The plasmid‐encoded *fosA8*, recently described in an *E*.* coli* isolate recovered from urine, showed the highest identity with a *fosA* gene identified in the chromosome of the enterobacterial species *Lecleria adecarboxylata*. This species is a rare source of infections in humans and is intrinsic resistant to fosfomycin probably due to high expression levels of the *fosA* ^LA^ gene (Poirel et al., 2019).

*FosA9* was first described in an *E*.* coli* isolate from a blood culture of a patient (ten Doesschate et al., 2019). The patient had a suspicion of chronic endovascular infection of the aortic bifurcation graft and had a history of recurrent episodes of sepsis with blood cultures yielding *Propionibacterium* spp., *Klebsiella variicola*,*Citrobacter koseri*, and *P*.* aeruginosa*. The patient also suffered a septic shock caused by *E*.* coli* which was treated with intravenous ceftriaxone. The treatment to suppress the chronic infection (amoxicillin/clavulanic acid and ciprofloxacin) was complemented with oral fosfomycin treatment since the *E*.* coli* isolate was resistant against the previously used antibiotic combination. Months later, *E*.* coli* with an identical resistance pattern could still be isolated from blood cultures, except for being additionally resistant to fosfomycin. Fosfomycin resistance could be traced back to the acquisition of a 3573 bp insertion including IS*Ecp1* and a *fosA* gene which was named *fosA9*. Genetic analysis of *fosA* revealed *K*.* variicola* to be the likely source of *fosA*9 acquired by *E*.*coli*. The authors hypothesized that *fosA9* was transferred from *K*.* variicola* to *E*.* coli* in the gastrointestinal tract since *K*.* variicola* was not co‐cultured in the blood at the time of *E*.* coli* bacteraemia (ten Doesschate et al., 2019).

FosA2 and FosA7 were reported as chromosomal variants of FosA of *E*.* cloace* and *S*.* enterica* serovar Heidelberg, respectively, and they would be named FosA^EC^ and FosA^SH^ in the newly proposed nomenclature (Ito et al., 2017).

The chromosomal progenitor of the recently reported plasmid‐mediated fosfomycin resistance genes *fosL1* and *fosL2* and the origin of *fosC2* is still unclear. FosC2 is a rarely described fosfomycin‐modifying enzyme identified on a conjugative plasmid in *E*.* coli* in Japan (Wachino et al., 2010). This gene was not yet detected in Europe.

## 3 PLASMID TYPES INVOLVED IN THE DISSEMINATION OF FOSFOMYCIN‐MODIFYING ENZYMES AMONG ENTEROBACTERIACEAE

The most common plasmid families in Enterobacteriaceae involved in the spread of antimicrobial resistance genes are IncA/C, IncL (previously designated IncL/M), IncF, IncI, IncH, and IncN (Carattoli, 2013; Rozwandowicz et al., 2018). In Europe, fosfomycin‐modifying enzymes were present on plasmids belonging to the IncF, IncN, IncA/C, IncHI2, and IncX1 family, whereas IncF is the predominant plasmid incompatibility type. Some *fosA*‐encoding plasmids were not typeable. An overview of *fosA* gene‐carrying plasmids from enterobacterial isolates in Europe is shown in Table 2. FosA3 is the most frequently found fosfomycin‐modifying enzyme worldwide (Yang et al., 2019). The dissemination of the *fosA3* gene is closely associated with that of the ESBL gene *bla*_CTX‐M_. In Europe, *fosA3* was described in *E*.* coli*‐producing CTX‐M‐15, CTX‐M‐55, CTX‐M‐14, CTX‐M‐3, CTX‐M‐65, CTX‐M‐9, CTX‐M‐27, CTX‐M‐125, and CTX‐M‐2. The predominant vehicles of *fosA3* are IncFII plasmids including F2:A‐:B‐, F33:A1:B1, and F33:A‐:B‐ (Baron et al., 2020; Benzerara et al., 2017a; Lupo et al., 2018; Mendes et al., 2016). IncF plasmids are limited by host range to the family of Enterobacteriaceae and are usually low copy number plasmids >100 kb in size (Carattoli, 2013). IncFII plasmids (F2:A‐:B‐, F33:A‐:B‐ and others) also play a key role in the dissemination of *fosA3* among clinical isolates, companion, and food animals in Asian countries (Ho, Chan, Lo, Lai, et al., 2013; Ho, Chan, Lo, Law, et al., 2013; Hou et al., 2012; Yang et al., 2014). Plasmids identified in Europe show highly similar molecular features even though a direct link could not be made in most cases.

**TABLE 2:** Characteristics of *fosA*‐carrying plasmids from enterobacterial isolates in Europe

Year	Country	Isolate	*fos* gene	Size [kb]	Inc type	Co‐located resistance genes^a^	Conjugation (frequency)	Surrounding element	Accession number	Reference
2011	Germany	1978‐1	*fosA3*	225	IncN	*bla*_CTX‐M‐65,_ *floR*, *aac(6′)*‐*lb3*, *sul2*	Yes (1.17 x 10^−2^)	n.a.	—	Freitag et al. (2018)
		1978‐4	*fosA3*	70	IncN	*bla*_CTX‐M‐125_, aph(3′)‐IIa	Yes (1.24 x 10^−1^)	ΔIS*Ecp1*‐*bla* **_CTX‐M‐125_**‐ΔIS*903B*‐*fosA3*‐*orf1*‐*orf2*	MG456909	
		1979‐1	*fosA3*	245	IncHI2	*bla*_CTX‐M‐14_, *floR*,*aac(3)*‐*IVa*,*sul2*,*tetA*(A)	Yes (7.09 x 10^−2^)	n.a.	—	
		RH‐1238	*fosA3*	187	IncA/C_2_	*bla*_NDM‐1_, *bla*_CMY‐16_,*sul1*,*sul2*,*strA*,*strB*, *aac(6*′*)*‐*Ib*,*aadA5*,*aphA6*,*tetA*(A),*mph(A)*,*floR*,*dfrA7*	Yes	*sul2*‐*strA*‐*strB*‐*tetR(A)*‐*tetA(A)*‐*floR*‐ISCR2‐IS*26*‐*fosA3*‐IS*26*‐ISCR3‐IS*26*‐*Mrx*‐*mph(A)*‐IS*6100*‐*sul1*‐*aadA5*‐*dfrA7*‐*intI1*	KR091911	Villa et al. (2015)
2011	France	27732	*fosA4*	145	IncF18:A‐:B1	*bla_CTX‐M‐55_*	Yes	n.a.	—	Lupo et al. (2018)
2012		9	*fosA3*	n.a	IncFII, Inc I1	n.a.	Yes	n.a.	—	Benzerara et al. (2017)
		12	*fosA3*	n.a.	IncFII, Inc I1	n.a.	Yes	n.a.	—	
2013		34248	*fosA3*	48	IncF33:A1:B1	*rmtB*,*bla*_CTX‐M‐55_	No	n.a.	—	Lupo et al. (2018)
		36	*fosA3*	n.a.	IncFII	n.a.	Yes	n.a.	—	Benzerara et al. (2017)
		19	*fosA3*	n.a.	IncFII	n.a.	Yes	n.a.	—	
		34	*fosA3*	n.a.	nontypable	n.a.	No	n.a.	—	
		24	*fosA3*	n.a.	IncFII	n.a.	Yes	n.a.	—	
2014		35	*fosA3*	n.a.	IncFII	n.a.	Yes	n.a.	—	
		39	*fosA3*	n.a.	IncFII	n.a.	Yes	n.a.	—	
2015		42	*fosA3*	n.a.	colE nontypable	n.a.	Yes	n.a.	—	
		20	*fosA5*	n.a.	IncN	n.a.	Yes	n.a.	—	
2014		ESBL96	*fosA3*	71	IncF33:A‐:B‐	*bla*_CTX‐M‐15_, *bla*_TEM‐1b‐like_	Yes	IS*26*‐*fosA3*‐*orf1*‐*orf2*‐*orf3*‐IS*26*	PRJNA608701	Baron et al. (2020)
2012	Portugal	C1728	*fosA3*	n.a.	F2:A‐:B‐	*bla*_CTX‐M‐15_	Yes	IS*26*‐*fosA3*‐*orf1*‐*orf2*‐*orf3*‐IS*26*	KT734860	Mendes et al. (2016 **)**
2012	Switzerland	R249	*fosL1*	40	IncX1	*catB3*	Yes (~10^−2^)	ΔIS*91*‐*like*‐Tn*7L*‐like‐*fosL1*‐*gnat*‐Tn7R‐like‐ΔIS91‐like	MN464149	Kieffer et al. (2020)
2013		376	*fosA8*	50	IncN	n.a.	Yes (~10^−5^)	Δ*sprT*‐*fosA8*‐*ΔsprT*	MN150127, PRJNA558667	Poirel et al. (2019)
		21‐13	*fosA3*	~230	nontypable	*bla*_CTX‐M‐65_, *aph(4)*‐*la*, *aadA1*, *aac(3)*‐*IVa*,*aph(3′)*‐*lc*, *floR*, *sul1*, *tetA*(A), *dfrA14*	n.a.	IS*26*‐*fosA3*‐*orf1*‐*orf2*‐*orf3*‐IS*26*	NAPP00000000	Hindermann et al. (2017)
		144‐13	*fosA3*	~230	nontypable	*bla*_CTX‐M‐65_, *aph(4)*‐*la*, *aadA1*, *aac(3)*‐*IVa*,*aph(3’)*‐*lc*, *floR*, *sul1*, *tetA*(A), *dfrA14*	n.a.	IS*26*‐*fosA3*‐*orf1*‐*orf2*‐*orf3*‐IS*26*	NAOX00000000	
2015		125‐15	*fosA3*	~230	nontypable	*bla*_CTX‐M‐65_, *aph(4)*‐*la*, *aadA1*, *aac(3)*‐*IVa*, *floR*, *sul1*, *tetA*(A), *dfrA14*	n.a.	?‐*fosA3*‐*orf1*‐*orf2*‐*orf3*‐IS*26* ^b^	NAPA00000000	

Abbreviations: kb, kilobase; n.a., not available.

^a^ Aminoglycoside resistance genes: *aac(6’)*‐*lb3*, aph(3’)‐IIa, *aac(3)*‐*IVa*,*strA*,*strB*,*aac(6’)*‐*Ib*,*aadA5*,*aphA6*,*rmtB*,*aph(4)*‐*la*, *aadA1*, *aac(3)*‐*IVa*,*aph(3’)*‐*lc*; broad/extended‐spectrum β‐lactamase genes: *bla*_CTX‐M‐65_, *bla*_CTX‐M‐125_, *bla*_CTX‐M‐14_, *bla*_CMY‐16_,*bla_CTX_*_‐_ *_M_*_‐_ *_55_*,*bla*_CTX‐M‐15_, *bla*_TEM‐1b‐like_; carbapenemases: *bla*_NDM‐1_; chloramphenicol/florfenicol resistance genes: *floR*,*catB3*; macrolide resistance gene: *mph(A)*; sulfonamide resistance genes: *sul1*,*sul2*; tetracycline resistance gene: *tetA*(A); trimethoprim resistance genes: *dfrA7*,*dfrA14*

^b^ Upstream elements of *fosA3* not available.

*FosA*‐encoding IncN plasmids varied in size (50–225 kb) and in the number of resistance genes they carry. The two *fosA3*‐positive IncN plasmids were found in sprouts and harbored *bla*_CTX‐M‐65_ and *bla*_CTX‐M‐125_ ESBL genes, respectively. IncN/ST8 co‐harboring *fosA3* and *bla*_CTX‐M‐65_ have been described in pig and duck samples from China in the early 2000 s (Hou et al., 2013).

In France, a *fosA5*‐carrying IncN plasmid was identified in a patient simultaneously with the original description of *fosA5* on an IncA/C background in China (Benzerara et al., 2017a; Ma et al., 2015). The *fosA5*‐variant was previously designated as *fosKP96* and has been described on 50 kb IncN plasmids in China in a urine and blood isolate, respectively (Ho, Chan, Lo, Lai, et al., 2013).

Recently, a novel distinct *fosA*‐variant, *fosA8*, was identified from a CTX‐M‐15‐producing *E*.* coli* isolate recovered from urine in Switzerland. Mating‐out experiments revealed the presence of *fosA8* on a self‐conjugative IncN plasmid that also carried a kanamycin resistance marker. In contrast to *fosA3*‐plasmids, this plasmid did not carry the *bla*_CTX‐M‐15_ ESBL gene identified in this isolate (Poirel et al., 2019).

In one isolate from sprouts, *fosA3* was located on a conjugative 245 kb IncHI2 plasmid. This plasmid additionally encoded genes for ESBL (*bla*_CTX‐M‐14_), florfenicol resistance (*floR*), aminoglycoside resistance (*aac(3)*‐*Iva*), sulfonamide resistance (*sul2*), and tetracycline resistance (*tetA*(A)) (Freitag et al., 2018). IncHI2 plasmids are usually large plasmids (>250 kb) and are frequently encountered in clinical enterobacterial isolates associated with the dissemination of relevant antimicrobial resistance genes (García‐Fernández & Carattoli, 2020). The first description of *fosA3* located on IncHI2 plasmids was reported from China among chicken isolates. An epidemic IncHI2/ST3 plasmid (~230 kb) drives the dissemination of *fosA3* along with *bla*_CTX‐14, CXT‐M‐15_, or_CTX‐M‐65_ in different geographic regions in China (Yang et al., 2014).

A novel plasmid‐mediated fosfomycin resistance gene, *fosL1*, was recently identified in a collection of ESBL‐producing *E*.* coli* isolates from Switzerland. The conjugative 40 kb IncX1 plasmid co‐harbored the *catB3* gene encoding a chloramphenicol acetyltransferase along with the fosfomycin resistance determinant. The authors performed an *in silico* analysis using the NCBI database and identified *fosL1* in a *Salmonella enterica* isolate from the United States. Furthermore, another putative glutathione‐S‐transferase FosL2 showing only five amino acid differences to FosL1 was found in a second *S*.* enterica* isolate. In contrast to the Swiss isolate, *fosL1* and *fosL2* were found to be located on IncQ1 and IncP‐like plasmids, respectively (Kieffer et al., 2020).

The wide dissemination of resistance genes is most often linked to horizontal gene transfer via plasmids, but also other mobile structures like transposons and insertion elements are important features when studying transmission pathways. IS*26* plays a key role in the dissemination and mobilization of fosfomycin‐modifying enzymes and numerous descriptions of IS*26* (composite) transposon‐like structures have been published. Integrons are important genetic elements for the acquisition of multiple resistance genes in bacteria. In contrast to mobile genetic elements, they are not considered to be mobile as such since they lack functions for self‐mobility. However, fosfomycin resistance determinants have been described within type 1 integrons on plasmids. Yang et al. have compiled surrounding elements of *fosA* genes in Enterobacteriaceae (Yang et al., 2019).

Co‐selection by other antimicrobials, especially third‐generation cephalosporins, and its localization on highly epidemic self‐transferable plasmids seem to play a major role in the spread of plasmid‐mediated fosfomycin resistance determinants. Furthermore, resistance genes against aminoglycoside (in particular *rmtB*), florfenicol (*floR*), or sulfonamide (*sul1*,*sul2*) are often co‐located with *fosA* genes.

## 4 MOBILE FOSFOMYCIN RESISTANCE GENES IN ENTEROBACTERIACEAE ISOLATED AT THE HUMAN–ANIMAL–ENVIRONMENT INTERFACE IN EUROPE

The first plasmid‐encoded fosfomycin‐modifying gene, *fosA*, was reported in clinical isolates of *Serratia marcescens* in 1980 in Spain (Mendoza et al., 1980). Nine fosfomycin‐resistant *S*.* marcescens* were studied in more detail and two types of fosfomycin resistance genes harboring conjugative plasmids were identified (Mendoza et al., 1980). In at least one case, *fosA* was associated with the insertion sequence IS*2921* in the composite transposon Tn*2921* (Navas et al., 1990). The results of a surveillance study performed fourteen years after the introduction of fosfomycin in Spain indicate that *fosA* is no longer confined to hospital environments since it could be detected in sewage samples in six out of seven provinces tested. Furthermore, *fosA* could be detected by hybridization in clinical isolates from three hospitals and the gene was carried by species of Enterobacteriaceae, *Pseudomonas* spp., and *Acinetobacter* spp. (Terán et al., 1988).

In the late nineties, an epidemiological multicentre survey was performed in Italy for which sixty out of 219 fosfomycin‐resistant bacteria selected from more than 7400 urinary pathogens were screened for *fosA* and *fosB*. In only three enterobacterial isolates (1 *Enterobacter* sp. and 2 *E*.* cloacae*), *fosA* could be detected by PCR (Arca et al., 1997). However, the location of *fosA* genes is uncertain since hybridization using undigested plasmid DNA failed. With the current state of knowledge, a possible chromosomal location of *fosA* is likely (Ito et al., 2017).

After these early publications, reports on mobile fosfomycin resistance in Europe were sparse. The renewed interest in fosfomycin as a potential treatment against MDR Gram‐negative bacilli and the first report of *fosA3* 2010 in Japan involving ESBL‐producing *E*.* coli* reactivated the awareness of mobile fosfomycin resistance (Giske, 2015; Wachino et al., 2010). Fosfomycin‐modifying enzymes, and especially FosA3, are meanwhile widespread in Asia among clinical isolates, pets, and livestock (Ho, Chan, Lo, Lai, et al., 2013; Ho, Chan, Lo, Law, Li, et al., 2013; Hou et al., 2012; Lee et al., 2012).

Characteristics of enterobacterial isolates encoding plasmid‐mediated *fosA* in Europe are summarized in Table 3. The first report of *fosA3* in Europe was in a *Salmonella enterica* serovar Corvallis strain isolated from a migratory wild bird in Germany. The fosfomycin resistance determinant was located on a large (187 kb) conjugative plasmid classified as IncA/C encoding *bla*_NDM‐1_, *bla*_CMY‐16_, *fosA3*,*sul1*,*sul2*,*strA*,*strB*,*aac(6’)*‐*Ib*,*aadA5*,*aphA6*,*tetA(A)*,*mph(A)*,*floR*,*dfrA7*, and *merA* genes, which confer resistance to most β‐lactam antibiotics including carbapenem, fosfomycin, co‐trimoxazole, aminoglycoside, tetracyclines, and macrolides. The authors propose a possible origin of the plasmid in the Asiatic region according to complete plasmid sequence analysis and the migration route of *Milvus migrans* which includes Europe and northern Asia during the summer (Villa et al., 2015). However, retrospective studies revealed that *fosA3* was already present in Europe at least since 2012 in clinical isolates in Portugal, France, and Switzerland, respectively (Benzerara et al., 2017; Mendes et al., 2016; Mueller et al., 2019). While the isolate from the patient in Portugal was associated with a urinary tract infection acquired after traveling to Asia (China, Philippines), there was no history of international travel in the patient population studied in France (Benzerara et al., 2017; Mendes et al., 2016). Of note, in the study of Mueller et al. (Mueller et al., 2019) two fosfomycin‐resistant *E*.* coli* isolates were identified which were tested negative for *fosA1*‐*A7* in PCR amplification although they exhibited an increased inhibition zone diameter on the agar plate in presence of FosA‐inhibitor sodium phosphonoformate. In two follow‐up studies, they identified three novel glutathione‐S‐transferase genes *fosA8*, *fosL1*, and *fosL2*, respectively (Kieffer et al., 2020; Poirel et al., 2019).

**TABLE 3:** Characteristics of enterobacterial isolates encoding plasmid‐mediated *fosA* in Europe.

Year	Country	Isolate	Source	Species	Phylogenetic group/MLST ST	Fosfomycin resistance gene	ESBL/carbapenemase	Resistance profile	Reference
2011	Germany	1978‐1	soy sprouts	*Escherichia coli*	A:ST10 (CC10)	fosA3	CTX‐M‐65	n.a.	Freitag et al. (2018)
		1978‐4 (pCTX48)	soy sprouts	*Escherichia coli*	A: ST542	fosA3	CTX‐M‐125	n.a.	
		1979‐1	soy sprouts	*Escherichia coli*	B1:ST527	fosA3	CTX‐M‐14	n.a.	
2015		RH‐1238	black kite	*Salmonella enterica* serovar Corvallis	n.a	fosA3	NDM‐1	n.a.	Villa et al. (2015)
2011	France	27732	monkey	*Escherichia coli*	B1:ST4380	fosA4	CTX‐M‐55	FOS, GM, NN, S, SMZ, TE, TMP	Lupo et al. (2018)
2012		9	urine	*Escherichia coli*	ST559 (CC10)	fosA3	CTX‐M‐55	n.a.	Benzerara et al. (2017)
		12	urine	*Escherichia coli*	ST559 (CC10)	fosA3	CTX‐M‐55	n.a.	
2013		34248	bovine	*Escherichia coli*	A:ST10 (CC10)	fosA3	CTX‐M‐55	AN, C, ENO, FOS, GM, K, NA, NET, NN, S, SMZ, TE, TMP	Lupo et al. (2018)
		36	blood	*Escherichia coli*	ST new	fosA3	CTX‐M‐55	n.a.	
		19	urine	*Escherichia coli*	ST new	fosA3	CTX‐M‐15	n.a.	
		34	urine	*Escherichia coli*	ST2015	fosA3	CTX‐M‐2	n.a.	
		24	urine	*Escherichia coli*	ST4508	fosA3	CTX‐M‐15	n.a.	
2014		35	urine	*Escherichia coli*	ST69	fosA3	CTX‐M‐15	n.a.	
		39	joint fluid	*Escherichia coli*	ST69	fosA3	CTX‐M‐15	n.a.	
		ESBL96	river	*Escherichia coli*	n.a.	fosA3	CTX‐M‐15	n.a.	Baron et al. (2020)
2015		42	urine	*Escherichia coli*	ST457	fosA3	CTX‐M‐14	n.a.	
		20	feces	*Escherichia coli*	ST new	fosA5	CTX‐M‐15	n.a.	
		RDP19	urine	*Escherichia coli*	ST219	fosA^a^	CTX‐M‐55	n.a.	Birgy et al. (2018)
2012	Portugal	C1728	urine	*Escherichia coli*	D:ST393	fosA3	CTX‐M‐15	AZM, CIP, CTX, FEP, FOS, GM, K, NET, NN, S, SMZ, TE, TMP	Mendes et al. (2016)
2017	Spain	n.a.	urine	*Escherichia coli*	A:ST10 (CC10)	fosA3	CTX‐M‐14	AM, CIP,CTX, CXM, FOS, GM	Loras et al. (2020)
		n.a.	urine	*Escherichia coli*	A:ST10 (CC10)	fosA3	CTX‐M‐27	AM, CTX, CXM, FOS, SMZ, TMP	
2012	Switzerland	R249	urine	*Escherichia coli*	B1:ST new	fosL1	CTX‐M‐15	C, CIP, CTX, FOS, K, NN, SMZ, TE, TMP	Kieffer et al. (2020)
		n.a.	patient	*Escherichia coli*	A:ST10 (CC10)	fosA3	CTX‐M‐3	n.a.	Mueller et al. (2019 **)**
2013		n.a.	patient	*Escherichia coli*	ST117	fosA3	CTX‐M‐3	n.a.	
		n.a.	patient	*Escherichia coli*	ST359 (CC101)	fosA3	CTX‐M9	n.a.	
		n.a.	patient	*Escherichia coli*	ST69	fosA3	CTX‐M‐15	n.a.	
		n.a.	patient	*Escherichia coli*	ST12	fosA4	n.a.	n.a.	
		376	urine	*Escherichia coli*	ST40	fosA8	CTX‐M‐15	CIP, CTX, FOS, K, NN, TE,	Poirel et al. (2019)
		21‐13 (N13‐0312)	urine	*Salmonella enterica* serovar Infantis	ST 32 variant	fosA3	CTX‐M‐65	AM, C, CF, CTX, FOS, GM, K, NA, S, SMZ, TE	Hindermann et al. (2017)
		144‐13 (N13‐2103)	food	*Salmonella enterica* serovar Infantis	ST 32	fosA3	CTX‐M‐65	AM, C, CF, CTX, FOS, GM, K, NA, S, SMZ, TE, TMP	
2015		125‐15 (N15‐1708)	food	*Salmonella enterica* serovar Infantis	ST 32	fosA3	CTX‐M‐65	AM, C, CF, CTX, FOS, NA, SMZ, TE, TMP	
2017	Croatia	18464	urine	*Escherichia coli*	n.a.	fosA*	OXA‐48	n.a.	Bedenić et al. (2018)
2019	Netherland	n.a.	blood	*Escherichia coli*	n.a	fosA9	None	n.a.	ten oesschate et al. (2019)

Abbreviations: AM, ampicillin; AN, amikacin; AZM, azithromycin; C, chloramphenicol; CC, clonal complex; CF, cephalothin; CIP, ciprofloxacin; CTX, cefotaxime; CXM, cefuroxime; ENO, enrofloxacin; ESBL, Extended‐spectrum beta‐lactamase; FEP, cefepime; FOS, fosfomycin; GM, gentamicin; K, kanamycin; MLST, Multi locus sequence typing; n.a., not available; NA, nalidixic acid; NET, netilmicin; NN, tobramycin; S, streptomycin; SMZ, sulfamethoxazole; ST, Sequence type; TE, tetracycline; TMP: trimethoprim.

^a^ fosA‐type not further analyzed.

In more recent studies, *fosA* genes were reported from clinical isolates from Croatia, France, and Spain (Bedenić et al., 2018; Birgy et al., 2018; Loras et al., 2020) but only in the study from Spain were fosfomycin‐resistant isolates explicitly investigated. Two isolates out of 39 randomly selected fosfomycin‐resistant *E*.* coli* from urine samples carried a plasmid‐borne *fosA3* gene and both were ESBL‐producer causing complicated UTI (Loras et al., 2020). The focus of the study from Croatia was the epidemiology, antibiotic resistance mechanisms, and routes of spread of OXA‐48 carbapenemase among clinical isolates collected in 10 hospitals. Within this study, three *fosA* harboring enterobacterial isolates were identified (2 *K*.* pneumoniae*, 1 *E*.* coli*) whereof the later was an OXA‐48 producing MDR UTI isolate (Bedenić et al., 2018). The report from France described the case of a 3‐year‐old boy originating from Bahrain who was hospitalized for pyelonephritis due to an ESBL‐producing *E*.* coli* infection. Whole‐genome sequencing has shown that the strain possesses a total number of seventeen antimicrobial resistance genes including the ESBL gene *bla*_CTX‐M‐55_, the glutathione‐S‐transferase gene *fosA*, the extended‐spectrum aminoglycoside resistance methylase gene *armA*, and the mobile colistin resistance gene *mcr*‐*1*. The combination of these resistance genes is particularly worrying because colistin and fosfomycin remain one of the last‐resort therapeutic options in the case of MDR Gram‐negative bacteria (Birgy et al., 2018).

Besides the description of a *fosA3*‐positive *S*.* enterica* serovar, Corvallis isolate from a migratory bird in Germany, mobile fosfomycin‐modifying genes have been described in animals in France. As part of a global survey of ESBLs in animals in France, the authors investigated a collection of 862 *E*.* coli* isolates suspected to produce an ESBL. Of those, CTX‐M‐55‐producing isolates were further studied for their co‐resistance to fosfomycin, aminoglycosides, fluoroquinolones, and colistin since *bla*_CTX‐M‐55_ is frequently co‐localized with resistance genes such as *fosA3*, plasmid‐mediated quinolone resistance genes, *mcr*‐*1* or −*3* and 16S rRNA methylases such as *rmtB*, a gene‐encoding pan‐aminoglycoside resistance (Lupo et al., 2018; Yang et al., 2014). One bovine isolate and one isolate from a monkey encoded *fosA3* and *fosA4*, respectively. *FosA3* was co‐located with *rmtB* and *bla*_CTX‐M‐55_ on a 48 kb IncF33:A1:B1 plasmid whereas *fosA4* was detected and co‐located with *bla*_CTX‐M‐55_ on a 145 kb IncF18:A‐:B1 plasmid. Of note, the monkey isolate (*fosA4*) was of Asian origin as this animal was introduced to France through a French animal centre (Lupo et al., 2018).

Also in France, ESBL‐harboring plasmids from *E*.* coli* isolated from river water were studied recently. Of the 21 randomly selected isolates, one plasmid (IncF33:A‐:B‐) contained the resistance determinants *bla*_CTX‐M‐55_, *bla*_TEM‐1b‐like,_ and *fosA* and was highly similar to a plasmid from an *E*.* coli* of chicken origin in China (Baron et al., 2020).

In the context of a study on ESBL‐encoding plasmids among *E*.* coli* isolates from fresh vegetables, three plasmids were found co‐harboring *bla*_CTX‐M_ and *fosA3*. The conjugative plasmids were found in *E*.* coli* isolates originating from soy sprouts from the Netherlands in 2011 (Freitag et al., 2018). The presence of multidrug resistance plasmids in *E*.* coli* isolates in vegetables underlines the importance of greens as a possible reservoir for resistance genes.

*Salmonella enterica* serovar Infantis has emerged as the fourth most common serovar causing human salmonellosis in Europe, and it is one of the most frequently reported serovar in broilers and broiler meat (EFSA, 2016). This serovar has been associated with a resistance and virulence megaplasmids (270‐320 kb) pESI (plasmid for emerging *S*.* enterica* serovar Infantis). One subgroup of this pESI‐like plasmids includes a multidrug resistance plasmid encoding up to ten different resistance genes including *bla*_CTX‐M‐65_ and *fosA3*. Highly similar plasmids have been described in a human isolate from Italy, along with the poultry production chain in the United States and one human and two food isolates in Switzerland, respectively (Franco et al., 2015; Hindermann et al., 2017; Tate et al., 2017).

Tracking the transfer dynamics of resistance plasmids is an almost impossible task. However, the data point out that *fosA3* was present in isolates of human, animal, food, and environmental origin. At the human–environment interface, *fosA3* was found to be located on the same plasmid backbone, that is, IncF. Surface waters are supposed to be putative reservoirs of multiresistant bacteria since they collect material from wastewater plants, water of urban or industrial effluents, and agricultural activities. Furthermore, wastewater has been described to reflect the current status of antibiotic‐resistant bacteria in the population (Kwak et al., 2015). IncN plasmids carrying *fosA3* seem to circulate among the human–food–animal–environment since such plasmids were described from all these origins. Other plasmid incompatibility types than IncF and IncN appear sporadically which makes tracking even more difficult.

This review has several limitations. First, fosfomycin resistance is not routinely tested in most monitoring and surveillance studies and if so, the resistance mechanism is often not further analyzed. Second, in many studies, fosfomycin resistance is not of main interest and is only detected by WGS and data analysis. These might cause underestimation of the current situation of the spread of mobile fosfomycin‐modifying enzymes.

## 5 CONCLUSIONS

The detection of mobile fosfomycin resistance genes in Enterobacteriaceae has increased in recent years. Certain *fosA* types (e.g., *fosA3*) are predominant and their occurrence is frequently linked to specific plasmids (e.g., IncFII family, IncN). Data from recent studies suggest that the therapeutic use of other antibiotic classes promotes the emergence and dissemination of fosfomycin‐resistant Enterobacteriaceae, since additional resistance genes are often present, first, and foremost extended‐spectrum beta‐lactamases. Several studies highlighted in this review provide evidence for the transmission of mobile fosfomycin resistance genes in Enterobacteriaceae at the human–animal–environment interface. The implementation of international and national mitigation strategies based on a One Health approach, including increased surveillance and monitoring of fosfomycin resistance in Enterobacteriaceae as well as the optimization of therapeutic strategies are necessary.

## CONFLICT OF INTEREST

None declared.

## AUTHOR CONTRIBUTION

**Katrin Zurfluh:** Conceptualization (equal); Data curation (lead); Writing‐original draft (lead); Writing‐review & editing (equal). **Andrea Treier:** Writing‐original draft (supporting); Writing‐review & editing (equal). **Kira Schmitt:** Writing‐original draft (supporting); Writing‐review & editing (equal). **Roger Stephan:** Conceptualization (equal); Funding acquisition (lead); Supervision (lead); Writing‐original draft (supporting); Writing‐review & editing (equal).

## ETHICS STATEMENT

None required.

## Data Availability

Not applicable.
